# ROS and the DNA damage response in cancer

**DOI:** 10.1016/j.redox.2018.101084

**Published:** 2018-12-21

**Authors:** Upadhyayula Sai Srinivas, Bryce W.Q. Tan, Balamurugan A. Vellayappan, Anand D. Jeyasekharan

**Affiliations:** a Cancer Science Institute of Singapore, National University of Singapore, Singapore; b Department of Radiation Oncology, National University Hospital, Singapore; c Department of Haematology-Oncology, National University Hospital, Singapore

**Keywords:** AML, Acute myeloid leukemia, ATM, Ataxia telangiectasia mutated, ATR, Ataxia telangiectasia mutated and Rad3 related, ATRIP, ATR interacting protein, BSO, Buthionine sulfoximine, BER, Base excision repair, Cdc25, Cell division cycle 25, DDR, DNA damage response, DNA PK, DNA-dependent protein kinase, dNTP, deoxyribonucleotide triphosphate, DSB, Double strand break, ETC, Electron transport chain, H_2_O_2_, Hydrogen peroxide, HER-2, human epidermal growth factor receptor 2, HR, Homologous recombination, ICD, Immunogenic cell death, MAPK, Mitogen-activated protein kinases, Mdm2, Mouse double minute 2, MRN, Mre11-Rad50-Nbs1, mtROS, Mitochondrial ROS, NADPH, Nicotinamide adenine dinucleotide phosphate, NCF2, Neutrophil Cytosolic Factor 2, NHEJ, Non-homologous end joining, NRF-2, Nuclear factor (erythroid-derived 2)-like 2, OXPHOS, Oxidative phosphorylation, PLK1, Polo-like kinase 1, PPP, Pentose phosphate pathway, ROS, Reactive oxygen species, RPA, Replication protein A, SOD, Superoxide dismutase, XRCC4, X-ray repair cross-complementing protein 4, Reactive Oxygen Species, ROS, DNA damage response, DDR, Chemotherapy, Radiotherapy

## Abstract

Reactive oxygen species (ROS) are a group of short-lived, highly reactive, oxygen-containing molecules that can induce DNA damage and affect the DNA damage response (DDR). There is unequivocal pre-clinical and clinical evidence that ROS influence the genotoxic stress caused by chemotherapeutics agents and ionizing radiation. Recent studies have provided mechanistic insight into how ROS can also influence the cellular response to DNA damage caused by genotoxic therapy, especially in the context of Double Strand Breaks (DSBs). This has led to the clinical evaluation of agents modulating ROS in combination with genotoxic therapy for cancer, with mixed success so far. These studies point to context dependent outcomes with ROS modulator combinations with Chemotherapy and radiotherapy, indicating a need for additional pre-clinical research in the field. In this review, we discuss the current knowledge on the effect of ROS in the DNA damage response, and its clinical relevance.

## 1 Introduction to the DNA damage response and ROS

### 1.1 Introduction to the DNA damage response

**Fig. 1:**
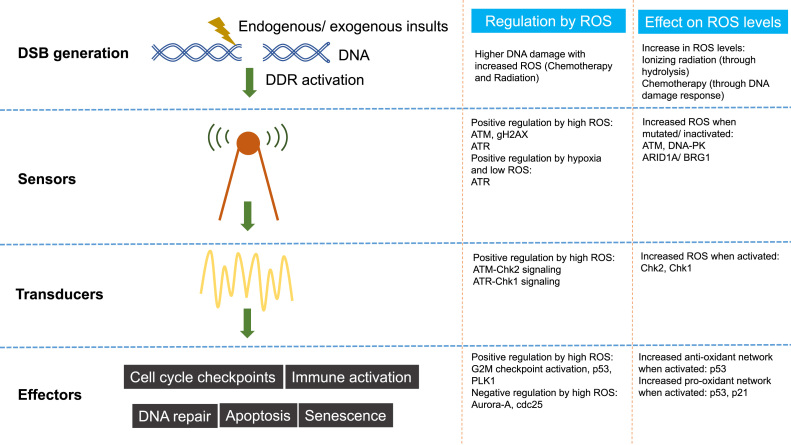
An overview of interactions between ROS and the DDR.

DNA damage refers to physical or chemical changes to DNA in cells, which can affect the interpretation and transmission of genetic information. DNA can be damaged by a variety of exogenous and endogenous insults including chemicals, radiation, free radicals, and topological changes, each causing distinct forms of damage [1]. Cells have evolved complex processes for dealing with damage to the genome. Depending on the nature of the lesion in DNA, specific pathways are activated to facilitate identification of the damaged regions and their repair [2], [3]. A particularly dangerous lesion is the DNA double strand break (DSB) which can be mutagenic due to chromosomal rearrangements or loss of genetic information due to erroneous DNA repair. In response to DNA damage a network of events collectively termed as the DNA damage response (DDR) is activated. This response includes DNA damage recognition, activation of checkpoints, cell cycle arrest, and eventually final outcomes of repair, apoptosis and immune clearance [4], [5]. The molecular components of the DSB induced DDR have been studied in detail, and are typically classified into three major groups - “sensors” which recognize damage, “transducers” which coordinate signaling, and “effectors” which mediate eventual outcomes (Fig. 1) [6]. Other DNA damage response/ repair pathways include Mismatch Repair (MMR) for mismatched bases, Base Excision Repair (BER) for base modifications, Nucleotide Excision Repair (NER) for intra-strand cross links and thymidine dimers, Single Strand Annealing (SSA) for single strand DNA (ssDNA) damage and Transcription coupled repair (TCR) for transcription associated damage [3]. The DDR leading from DSBs on the other hand activate a network of related pathways including Homologous Recombination (HR), Non-Homologous End Joining (NHEJ), Microhomology Mediated End Joining (MMEJ) and the Fanconi Anaemia (FA) repair complex [3]. Amongst these, the influence of ROS on the DSB induced DDR pathway will be the topic of this review. The effect of ROS on other DNA damage response pathways (especially oxidative damage) has been extensively reviewed elsewhere [7]. The response to DSBs is particularly relevant in carcinogenesis and cancer therapy, as many of the components of the pathway are mutated in cancer, and most current cancer treatment (chemotherapy and radiotherapy) exploits these defects [8].

### 1.2 Introduction to ROS

Reactive oxygen species (ROS) comprise of a family of short-lived molecules like O_2_^-^, H_2_O_2_ and •OH, first described in skeletal muscle as free radicals [9]. Though initially thought to be a hazardous byproduct of mitochondrial respiration, discoveries in the last four decades have illuminated functional roles for ROS in cells- from aiding immunity (e.g. oxidative bursts in phagocytes to eliminate pathogens) [10] to acting as signaling molecules (e.g·H_2_O_2_ regulating NFκB, MAPK pathways) [11]. ROS are produced endogenously by (i) mitochondria (where O_2_ acts as a terminal electron acceptor for electron transport chain) [12], (ii) NADPH oxidase, a cell membrane bound enzyme [13], (iii) Peroxisomes (which contain enzymes that produce H_2_O_2_ e.g. polyamine oxidase) [14], (iv) Endoplasmic reticulum (produce H_2_O_2_ as a byproduct during protein folding); or upon exposure to exogenous stress like ionizing radiation (IR), chemotherapeutic drugs and environmental insults, which affect the organelles and enzymes listed above [15].

ROS production has been implicated in mediating chemotherapy or radiotherapy responses via its effects on downstream cell survival or death signaling cascades [16], [17], [18]. This has led to suggestions that ROS modulators could be used for cancer primary prevention, or to enhance therapeutic responses [18], [19]. However, there has been little progress in translating ROS knowledge from labs to the clinic. For example, despite promising in-vitro data, most antioxidant trials in cancer prevention have yielded negative results [20], [21], highlighting the need for additional basic understanding of this process in cells. This review aims to examine mechanisms by which ROS mediates the DNA damage response, and provide insights for clinical exploitation of ROS in cancer.

## 2 Role of ROS in modulating the DNA damage response

### 2.1 The role of ROS in the induction of DNA damage

#### 2.1.1 Role of ROS in mediating genotoxin induced damage

ROS are well recognized as mediators of DNA damage. For example, Ionizing Radiation (IR) induces DSBs through direct high-energy damage to the sugar backbone of DNA, but also through free radicals generated in cells- mostly •OH from water [22]. Chemotherapeutics like doxorubicin and cisplatin increase ROS levels, which contributes to their genotoxicity [23], [24]. ROS have also been reported to directly induce other forms of DNA damage through oxidizing nucleoside bases (e.g. formation of 8-oxo guanine) [25], which can lead to G-T or G-A transversions if unrepaired. Oxidized bases are typically recognized and repaired by the BER pathway, but when they occur simultaneously on opposing strands, attempted BER can lead to the generation of DSBs [26]. ROS accumulation also induces mitochondrial DNA lesions, strand breaks and degradation of mitochondrial DNA [27].

#### 2.1.2 Role of ROS in DNA damage by oncogenic replication stress

An important source of endogenous DNA damage and DSB generation in cancer is oncogene induced replication stress [28]. Proto-oncogenes aid in cell growth and proliferation, but mutations or overexpression can transform them into oncogenes that drive continuous cell growth and carcinogenesis. Oncogenic cell cycles are typically associated with replication stress, which is defined as aberrant replication fork progression and DNA synthesis [29]. Replication stress ultimately results in genomic instability and paves the way for tumor development through the accumulation of additional pro-carcinogenic changes [28], [30]. The DDR acts as a barrier which limits the expansion of abnormally replicating cells, and this leads to a selective pressure for DDR defects in carcinogenesis [31].

Replication stress arises from a variety of sources including aberrant origin firing, decoupling of DNA polymerase-helicase activity, and physical obstacles to the replication fork [29]. Oncogene activation leads to an increase in ROS, which in turn influences the occurrence of replication stress [32], [33]. ROS oxidize dNTPs to affect polymerase activity and thereby reduce replication fork velocity in vitro [34], [35]. ROS can also affect replication fork progression through dissociation of peroxiredoxin2 oligomers (PRDX2). PRDX2 forms a replisome associated ROS sensor that binds to TIMELESS, a fork accelerator. Elevated ROS lead to dissociation of PRDX2 and TIMELESS, thus slowing replication fork speed [36]. Oxidized bases occurring from ROS activity also present a physical obstacle to replication forks [37], resulting in the breakdown of replication forks at fragile sites across the genome. Fork breakdown leads to DSBs and ultimately under-replicated or over-replicated DNA [28], with concomitant genomic instability in the tumor. Modulation of replication stress by ROS has clinical implications, with the development of several agents- notably ATR and WEE1 inhibitors, which target replication stress in tumours [28].

### 2.2 The role of ROS on sensing of DSBs

#### 2.2.1 Sensor kinases

The initial sensing of DSBs is performed by the kinases ATM/ ATR and DNA-PK, along with a network of sensor proteins [38], [39]. ATM loss, which is common in cancer, leads to an increase in ROS. This elevation in ROS appears unrelated to the canonical role of ATM in the DNA damage response. ATM loss modulates mitochondrial turnover, with an increase in aberrant mitochondria and therefore ROS [40], [41]. ATM-deficient cells also have increased ROS due to defects in NRF2 activity, a transcriptional factor which promotes the expression of antioxidant proteins under conditions of cellular stress [42], [43]. Accordingly, inhibition of the ATM-G6PD axis exacerbates mitochondrial oxidative stress and confers synthetic lethality with FLT3 tyrosine kinase inhibitors in AML [44]. Similarly, DNA-PK deficient cells accumulate higher ROS upon oxidative stress [45], [46].

The sensor kinases however can also directly be modulated by ROS levels, with distinctions between members of the family. ATM can be directly activated by oxidative stress, for example by H_2_O_2,_ leading to its autophosphorylation and subsequent downstream activation of the DDR pathway [47]. On the other hand ROS accumulation inhibits DNA- PKcs activity by altering its interaction with KU70/80 [45]. Oxidative stress by H_2_O_2_ requires ATR for γ-H2AX accumulation and activation of the DDR [48], as well as ATR dependent phosphorylation of Chk1 [49]. Further studies are needed to explore the effect of ROS on the activity of ATR, as well as the effect of clinical-grade ATR inhibitors on cellular ROS levels. Overall, the DDR sensor kinases appear to act to prevent ROS accumulation and protect the genomic integrity, although there are likely to be context specific variations depending on cell type and nature of insult.

#### 2.2.2 Chromatin remodelers

Brahma-related gene 1 (BRG-1) associated factor complex (BAF) are chromatin remodelers commonly mutated in cancer [50], and have a recently described role in the initial activation of the DNA damage response by modulating ATR activation [51], [52]. Two main components of BAF complex are AT-rich interacting domain 1 A (ARID1A) and BRG1, ATPase of the BAF complex. ROS lowers ARID1A expression by promoter methylation in ovarian cancers [53], [54], and ARID1A loss sensitizes ovarian cancer cells to ROS inducing agent elesclomol [55]. Importantly, ARID1A/BRG-1 loss increases reliance on OXPHOS, causing increased ROS, and synergizes with inhibitors of OXPHOS [56], offering a possible redox based therapeutic strategy for cancers harboring SWI/SNF mutations.

Histone H2AX is another chromatin factor that has been extensively studied in the DNA damage response [57]. Phosphorylated H2AX (γH2AX) helps to recruit multiple components of the DDR to the site of DNA DSBs to initiate DNA DSB repair [58], [59]. Deficiency of H2AX in-vivo is characterized by genomic instability and radiosensitivity [60], [61] arising from an impaired DDR. Interestingly, chronically elevated ROS mediates H2AX protein degradation, which is associated with decreased γH2AX and therefore improved sensitivity to platinum therapy in triple negative breast cancer [62]. Conversely, acute oxidative stress increases γH2AX activation and DDR signaling [63]. This has been suggested to blunt the treatment response to chemotherapy and radiation, and is associated with worse outcomes for colorectal [64], breast [65], and lung cancer [66]. The link between H2AX and ROS is bidirectional. γH2AX mediated activation of the Nox1-Rac1 complex [67], [68] regulates ROS production [69]. However, the pathophysiological relevance of γH2AX-mediated ROS production remains unclear.

### 2.3 Effect of ROS on signal transduction within the DDR

Downstream of the sensor kinases are the transducer kinases Chk2 (activated by ATM) and Chk1 (activated by ATR), which phosphorylate and regulate proteins involved in DDR, DNA repair and cell cycle arrest. Menadione and camphorquinone induced ROS accumulation increases phosphorylated Chk2 [70], [71]. N-acetylcysteine, an antioxidant reverses the synergistic effect between Chk2 inhibition and gemcitabine in pancreatic cancer cells highlighting the importance of ROS in activation of Chk2 [72].

Elevated levels of ROS also activate the ATR-Chk1 axis [34]. This is associated with poorer outcomes in breast cancer independent of hormonal status [73], and can mediate chemotherapy resistance in bladder cancer cells [74]. Accordingly, attenuation of ROS or ATR-Chk1 signaling confers chemosensitivity in platinum-resistant ovarian cancer cell lines with elevated levels of ROS [34]. Chk1 inhibition potentiates the cytotoxic effects of DNA-damage therapeutics in preclinical studies [75], [76], [77], although the relevance of ROS in this context has not been clearly defined. The ATR-Chk1 axis is a promising therapeutic target in cancer, and ROS dependent mechanisms that lead to ATR-Chk1 inhibitor resistance are worthy of further investigation.

### 2.4 Effect on cell cycle progression

Cell cycle arrest is an important aspect of the DDR, preventing cells with DNA damage from proceeding with cell division. In Hela cells, asperlin induced ROS leads to an ATM-Chk2 mediated G2/M arrest [78]. Similarly, ROS induced Chk1 activation leads to a p53 independent G2/M arrest in colorectal cancer cells [79]. Apart from their effects on the activation of cell cycle checkpoint proteins, ROS also promote cell cycle arrest by direct actions on the Cdc25 family of protein phosphatases (Cdc25A, B and C). The Cdc25 phosphatases promote cell cycle progression by removing inhibitory phosphates on cyclin dependent kinases (CDK) [80], and their levels/ activity are influenced by ROS. For example, ROS decreases Cdc25C protein levels to induce G2/M arrest [81]. Caulibugulone A (a family of isoquinoline quinones) induces ROS and reduces total Cdc25A levels [82]. Similarly, 17*β*-Oestradiol-induced ROS increases Cdc25A oxidation and reduces its phosphatase activity [83].

Mitotic entry and recovery from the G2/M arrest upon completion of DNA repair is mediated by the mitotic kinases Polo-like kinase 1 (PLK1) and AURORA-A. These kinases are frequently overexpressed in cancer and are also of interest in the context of ROS. PLK1 phosphorylates glucose-6-phosphate dehydrogenase, causing an increased PPP flux and production of NADPH, thereby increasing the antioxidant capacity of a cell. Interestingly, oxidative stress with H_2_O_2_ increases PLK1 expression in a p53 dependent manner [84], [85], but maintains a G2/M arrest. In contrast, ROS accumulation inhibits Aurora kinase A [86], even though PLK1 and Aurora-A are epistatic in the pathway. PLK1 and Aurora-A kinase inhibitors are currently in clinical trials, and understanding the dichotomous relation between ROS and these proteins may have clinical applications.

### 2.5 p53 transcriptional response, and apoptosis

p53 is a well-studied tumor suppressor that is mutated in over 50% of all cancers [87], [88], and affects multiple cellular responses to DNA damage. Upon cellular stress and DNA damage; p53 is stabilized and aids in transcription of genes to determine cell fate [89]. p53 is a redox protein with clusters of cysteine residues that can be targets of ROS [90], but it can also regulate ROS in turn [91]. Furthermore, ROS accumulation has different effects on cell fate depending on p53 status; with more apoptosis in cells with functional WT p53 [92]. p53 has an important role in regulating pro and antioxidant genes depending on ROS intensity [91]. With lower ROS intensity, p53 activates antioxidant genes, while with higher ROS intensity it switches on pro-oxidant genes [93]. In response to ROS production under basal cellular conditions, p53 upregulates transcription of several antioxidant genes including manganese superoxide dismutase (MnSOD), glutathione peroxidase 1 (Gpx1), Sestrins, Glutaminase 2 (GLS2), and TIGAR, which increase PPP and NADPH production [94], [95]. However, drastic increase of cellular ROS, for example by inhibition of thioredoxin reductase, an essential component of the thioredoxin antioxidant system, leads to JNK-mediated p53 activation and its downstream upregulation of pro-oxidant genes PUMA and PIGs [96]. Furthermore, under conditions of high ROS, p53 has been demonstrated to downregulate antioxidant proteins including SOD2 [97] and the anti-oxidant transcriptional factor Nrf2 [98]. This duality in p53 function with ROS intensity may decide the cell fate, with the protective arm of p53 activating processes to reduce cell stress with lower ROS intensity, while higher ROS intensity tips the balance towards cell death.

### 2.6 DNA repair

DNA repair is one of the effector outcomes of the DDR, but ROS so far has not been shown to affect DSB repair protein function directly. R-loops are DNA-RNA hybrids formed during replication-transcription conflicts in cells, and are a major source of genomic instability, requiring HR for resolution [29]. ROS induced R-loops are shown to require transcription coupled homologous recombination repair to protect actively transcribed genes in a Rad52 dependent manner [99]. ROS is typically implicated in regulating other DNA repair pathways such as BER, where the DNA glycosylase OGG1 is inhibited by ROS [100]. As 8-Oxo-dG can be potentially converted to DSBs, further work will be required to understand the contribution of ROS to DSB generation through this route. With clinical implications of interfering with DNA repair pathways becoming apparent, the direct effect of ROS on DNA repair proteins and its consequence in tumor development and chemo-resistance warrant more studies.

## 3 Clinical relevance of ROS, chemotherapy and radiotherapy responses

### 3.1 Cell death/ resistance in response to chemotherapy and radiation

Resistance to chemotherapy is a commonly encountered problem in clinical oncology, leading to disease recurrence and poor outcomes. Chemotherapeutic agents such as platinum derivatives and gemcitabine upregulate ROS in vitro [101], [102], [103], adding to their genotoxic effects. In addition to generating nuclear DNA adducts, platinum drugs increase mtROS via formation of mitochondrial DNA adducts [104], [105], [106], the extent of which correlates with cytotoxicity [24], [107]. Pro-oxidant strategies could therefore serve as adjuncts to improve the efficacy of chemotherapy and reduce the development of resistance [108]. For example, depletion of intracellular glutathione (GSH) using RNAi against the anti-oxidant transcription factor Nrf2 leads to increased ROS and increased sensitivity to chemotherapy in preclinical studies [103].

Radiotherapy using ionizing radiation (mega-voltage X-ray beams) is a widely used modality in cancer treatment. DNA damage can occur directly as the beam interacts with DNA strands in the nucleus, or indirectly via generation of free-radicals within the cell. The indirect method, accounting for about 80% of DNA damage, occurs when hydroxyl free radicals (•OH) are produced from the radiolysis of water molecules [109]. These molecules are able to diffuse a short distance into the nucleus to cause DNA damage. Antioxidant molecules within cells therefore can reduce the ability of ionizing radiation to cause DNA damage. Early studies observed that depletion of GSH could enhance radiosensitivity of squamous cell carcinoma cell lines [110]. More recent work has described the role in radio-resistance for Nrf2. Nrf2 is normally degraded via its interaction with a repressor protein Keap1. Decreased Keap1-Nrf2 interaction [111], [112] and loss-of-function mutations of Keap1 [112], [113] lead to aberrant Nrf2 activation, and therefore resistance to radiotherapy. Other mechanisms conferring radio-resistance include regulation of antioxidants by the synergistic effects of thioredoxin and GSH [114]. Cancer stem cells have active ROS-scavenging mechanisms and consequently show lower ROS levels and, less DNA damage from radiation, and therefore more radio-resistance [115].

### 3.2 Immunogenic cell death (ICD) after chemotherapy and radiation

ICD is increasingly appreciated as an important mechanism of chemotherapy mediated tumor cell-kill, where chemotherapy induced antigen release, immune priming and activation triggers an immune response against the tumor. The initial stages of immunogenic cell death are mediated by release of factors such as High-mobility group box 1 (HMGB1) protein and Calreticulin, and subsequent activation of the adaptive immune system through antigen presenting cells, leading to eventual T-cell mediated killing [116].

HMGB1 is a non-histone chromatin protein, which is released by dying cells into the micro-environment, where it plays a vital role in dendritic cell licensing and maturation. HMGB1 is a redox sensor, with cysteine 106 (Cys106) particularly important for the regulation of pro-inflammatory cytokine release [117], [118]. Reduction of three cysteine residues (Cys23, Cys45, and Cys106) induces chemotaxis of inflammatory cells [119], while oxidation of all three cysteines abolishes its pro-inflammatory and chemotactic properties [120]. As with other components of the DDR, the relationship between ROS and HMGB1 release is bi-directional. The antioxidant N-acetylcysteine attenuates HMGB1 release [121], and HMGB1 release in itself increases ROS production [122], which can lead to further oxidation of HMGB1. Oxidized HMGB1 enhances apoptosis and chemosensitivity in pancreatic and colorectal cancer cell lines [123], whereas reduced HMGB1 promotes autophagy-mediated chemoresistance towards melphalan, oxaliplatin and paclitaxel [123], [124]. Oxidized HMGB1 in apoptotic cells has however been reported to also mediate immunological tolerance [125], although the relevance of this finding to ICD after chemotherapy is unclear. Further studies are required to clarify the role and mechanisms underlying ROS-regulation of HMGB1 and its effects on in vivo tumor responses to chemotherapy [126].

ROS plays an active role in the pathways involved in immunogenic cell death including the induction of autophagy [127], and antigen presentation by immune cells [128], [129]. Induction of autophagy via increased levels of ROS results in biochemical hallmarks of ICD evasion [130]. Similarly, irradiation of necrotic high-grade gliomas increases the anti-tumor efficacy of dendritic cell (DC) vaccines, presumably via elevated levels of carbonylated proteins [131]. This suggests that ROS modulators could potentially play an important role in DC vaccine development and as adjuncts with other forms of immunotherapy, further highlighting its clinical relevance.

### 3.3 Combination studies with genotoxic agents in cancer

Modulators of ROS and redox pathways have been tested in combination with chemotherapy in clinical trials with mixed efficacy (summarized in Table 1). For instance, a single arm trial of NOV-002 (a formulation of disodium glutathione disulfide) in combination with standard neoadjuvant chemotherapy (AC-T) for stage II-IIIc HER2-negative breast cancer showed promising pCR rates [132], whereas phase 3 trial data on NOV-002 in non-small cell lung cancer has been disappointing [133]. While the pro-oxidant molecule Imexon demonstrated initial promising results in advanced pancreatic cancer in combination with gemcitabine [134], a larger phase II trial demonstrated no significant survival benefit or responses (ClinicalTrials.gov; NCT00637247). However, it showed single-agent clinical activity in refractory non-Hodgkin B-cell lymphoma [135], and will need to be further evaluated with chemotherapy in this setting. On the other end of the spectrum, due to the diverse effects described above for ROS in activating various DNA damage responses, high ROS is also associated with resistance to chemotherapy [18], [136]. Antioxidants such as ascorbate have been tested in this setting. However most of the clinical data on ascorbate-chemotherapy combinations are not randomized [137], and further RCTs are required to determine the efficacy of these strategies.

**Table 1:** Summary of clinical studies on ROS modulators in malignancies.

**Compound**	**Malignancies**	**Study construct**	**Outcomes**	**Reference**
**Pro-oxidants**				
NOV-002	Stage II to IIIc breast cancer	Phase 2; adjunct to doxorubicin-cyclophosphamide regimen, followed by docetaxel	Complete pathological response in 38%	[132]
	Advanced non-small cell lung cancer	Phase 3, randomized controlled trial: paclitaxel and carboplatin with NOV-002 vs placebo	No significant difference in overall survival	[141]
	Chemotherapy (platinum)-resistant ovarian cancer	Phase I, single arm: in combination with carboplatin	Progression-free survival of 14 weeks	[142]
Imexon	Advanced pancreatic adenocarcinoma	Phase I, single-arm trial; Phase II, randomized controlled trial	Phase I: partial response in 11%, 48% with stable disease; Phase II: Objective response in 13.2% (imexon arm), and 16.4% (placebo arm)	[134]
	Advanced prostate, breast, and non-small cell lung cancer	Phase I, single-arm: combination with docetaxel	4 out of 22 subjects with partial response, 11 with stable disease	[143]
BSO	High-risk neuroblastoma	Phase I, single-arm: combination with L-PAM	7 out of 25 patients with partial or mixed response	[144]
	Paediatric recurrent neuroblastoma	Phase I, single-arm: combination with L-PAM	18% response rate in 32 patients	[145]
Motexafin Gadolinium (MGd)	Brain metastases	Phase III, randomized-controlled trial: whole brain radiotherapy ± MGd	554 patients: Group with MGd had longer time to neurological progression (15 months versus 10 months)	[146]
	Intrinsic Pontine glioma	Phase II, single-arm: Radiotherapy + MGd	60 patients. 1-year OS: 53%; not significantly different from historical controls	[147]
	Non-small cell lung cancer	Phase I, dose-escalation study: MGd in combination with docetaxel and cisplatin; Phase II recruiting patients with previous platinum-based treatment: MGd in combination with pemetrexed	Phase I: 70% with partial response or stable disease; Phase II: No significant difference in progression-free survival or overall survival	[148]
**Antioxidant**				
Ascorbate	Advanced stage 3 or 4 ovarian cancer	Randomized controlled trial; carboplatin and paclitaxel in combination with ascorbate	16.75 months vs 25 months overall survival	[18]
	Advanced Pancreatic Cancer	Phase I/IIa trial; ascorbate with gemcitabine	Overall survival is 15.1 months, significantly higher than 5 months (no numerical value given) in placebo arm	[149]
	Metastatic pancreatic cancer	Phase I single-arm trial: combination with gemcitabine and erlotinib	7/9 subjects have stable disease	[150]
	Locally advanced pancreatic cancer	Phase I: Gemcitabine + 50 Gy RT + IV ascorbate	14 patients: OS 21.7 months (vs 12.7 months institution mean)	[151]

Further clinical research on ROS needs to take certain concepts into account. Firstly, in the context of a defined genotoxic agent in a particular cancer, identifying the specific ROS species involved in a) generation of DNA damage and b) in modulating the downstream DDR, would help in identifying specific therapeutic targets. Indeed, perturbation of redox status with a pan-antioxidant or pro-oxidant would have profound effects on both pro-survival and pro-death pathways [138], and may result in attenuation of specific chemotherapeutic responses [139]. Secondly, ROS has a dose dependent effect on activity of proteins leading to differential downstream outcomes [140], which are distinct in the setting of exogenous and endogenous ROS, and need to be evaluated in phase 1 dose finding studies with appropriate pharmacodynamic/ pharmacokinetic readouts. There is a clear need for further research outlining how chemotherapy and radiotherapy related DNA damage responses are influenced by ROS and ROS modulating drugs, using established and validated pre-clinical models.

## 4 Concluding remarks

The role of ROS in DNA damage response is multifaceted and pleomorphic. A distinction is required between oxidative stress leading to DNA damage/ downstream activation of DDR, and the role of ROS in modulating components of the DDR (signaling and effectors). There is compelling evidence that dysregulation of ROS contributes towards cancer pathogenesis as well as chemoresistance and radio-resistance, in a context specific manner. However, the modest responses of existing pan-antioxidant or pro-oxidants in advanced cancers could suggest that approaches aimed to reduce or increase ROS may not suffice. Future research on the specific mechanisms in chemo/radioresistance that are mediated by distinct reactive oxygen species, in distinct cellular contexts, will be valuable towards the development of drugs targeting these mechanisms.
